# Surgical Treatment of Single Pontomedullary Junction Metastasis from Lung Cancer

**DOI:** 10.1155/2022/4041506

**Published:** 2022-05-31

**Authors:** Paolo Missori, Simone Peschillo, Angela Ambrosone, Antonio Currà, Sergio Paolini

**Affiliations:** ^1^Department of Human Neurosciences, Neurosurgery, Policlinico Umberto I, “Sapienza” University of Rome, Italy; ^2^Department of Neurosurgery, University of Catania, Catania, Italy; ^3^Department of Medical-Surgical Sciences and Biotechnologies, Academic Neurology Unit, Ospedale A. Fiorini, Terracina, LT, “Sapienza” University of Rome, Polo Pontino, Italy; ^4^IRCCS Neuromed-Pozzilli, “Sapienza” University of Rome, Polo Pontino, Italy

## Abstract

**Background:**

When lung cancer develops a solitary metastasis at the pontomedullary junction, due to surgical risk, the current oncologic treatment is radiosurgery and chemotherapy. *Case Description*. We describe a patient with a single intrinsic metastasis at the pons and medulla. Removal was successful, without complication.

**Conclusion:**

Surgery can provide excellent results, and in selected patients, it should be considered a first-line treatment in experienced hands.

## 1. Introduction

Solitary metastases from lung cancer are very rarely found in the medulla oblongata and pons. In this location, autopsy studies have shown single or multiple metastases, which account for 0.06 to 7%, respectively [1]. Currently, the suggested oncologic treatment for this type of metastasis is radiosurgery and chemotherapeutic treatment [2]. Surgery has only been performed in exceptional cases. One study reported a subtotal excision of an intrinsic metastatic adenocarcinoma at the pontomedullary junction [3]. Two other studies have described surgical treatments for patients with lateral pontine and medulla oblongata metastases from adenocarcinoma [4, 5]. Here, we present the surgical removal of an intrinsic pontomedullary junction metastasis from lung cancer and its clinical course.

## 2. Case Presentation

**Figure 1:**
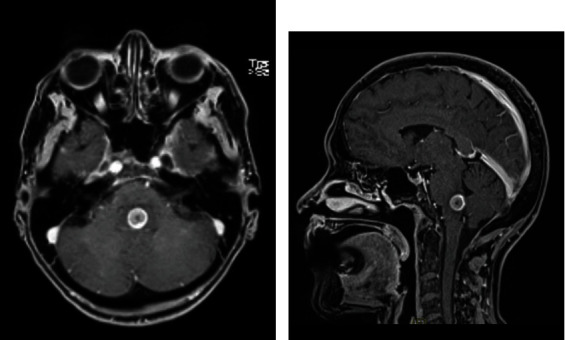
(a) Preoperative contrast-enhanced MRI shows a 13 mm intrinsic, roundish lesion in the posterior pontomedullary junction. (b) The floor of the inferior IV ventricle is slightly raised, and the tumor slightly protrudes from it.

**Figure 2:**
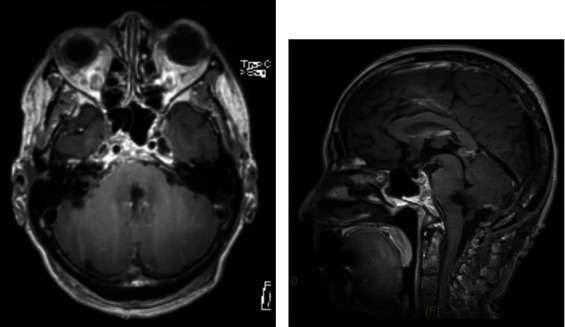
(a) Postoperative contrast-enhanced MRI confirms that the metastasis was totally removed. (b) The remaining medulla and pons tissues have filled the space, except for a very thin gap.

A 63-year-old female with a history of smoking was admitted to the hospital due to double vision and impaired balance, 2 weeks prior to our examination. On examination, she displayed severe, unsteady gait, left cranial nerve VI palsy, and reduced sensitivity to light touch and a pinprick in the right arm. CT and MR imaging studies showed a single 13 mm, roundish, intrinsic lesion at the pontomedullary junction (Figure 1). A chest X-ray showed a lesion in the left lung. A full-body CT scan excluded any other tumor location. Due to the rapid worsening of symptoms, surgical treatment was recommended. Through a suboccipital craniotomy and telovelar approach, we observed that the lesion slightly emerged from the IV ventricle floor. The lesion was completely removed (Figure 2). The histological diagnosis was adenocarcinoma. The postoperative course was uneventful, and the patient also underwent removal of the lung tumor. Due to T3 N1 M1b staging, a course of radiation and chemotherapeutic agents were administered. At an 8-month follow-up, the neurological examination showed persistent left VI nerve palsy and slight gait unsteadiness.

## 3. Discussion

Gamma knife radiosurgery is widely considered the best option for managing intrinsic brainstem metastases. However, adverse radiation effects are a major concern. Although this surgery is the treatment of choice for patients with multiple secondary brain lesions, surgery can be considered for patients with a single intrinsic brainstem lesion. Three mandatory points must be assessed for this treatment: first, the primary tumor must be considered entirely removable with the most suitable treatment; second, no other secondary localizations should be detected in the whole-body CT studies; and third, come out of the intrinsic brainstem metastasis. When pontomedullary junction metastases meet these criteria, surgical removal may be feasible in selected patients, without complications. Finally, the surgeon must be confident with the surgical approach, which takes place in a cisternal subarachnoid corridor; this is the last, but not least, criterion that is essential for performing a surgical treatment in patients with cancer.

## Data Availability

Raw data were generated at the Policlinico Umberto I, “Sapienza” University of Rome Hospital. Further enquiries can be directed to the corresponding author.
